# Proteome Analysis of Swine Macrophages after Infection with Two Genotype II African Swine Fever Isolates of Different Pathogenicity

**DOI:** 10.3390/v14102140

**Published:** 2022-09-28

**Authors:** Elisabeth Wöhnke, Gwenny Cackett, Finn Werner, Sandra Blome, Thomas C. Mettenleiter, Axel Karger

**Affiliations:** 1 Institute of Molecular Virology and Cell Biology, Friedrich-Loeffler-Institut, Federal Research Institute for Animal Health, Südufer 10, 17493 Greifswald, Germany; 2 Institute for Structural and Molecular Biology, Darwin Building, University College London, Gower Street, London WC1E 6BT, UK; 3 Institute of Diagnostic Virology, Friedrich-Loeffler-Institut, Federal Research Institute for Animal Health, Südufer 10, 17493 Greifswald, Germany; 4 Friedrich-Loeffler-Institut, Federal Research Institute for Animal Health, Südufer 10, 17493 Greifswald, Germany

**Keywords:** African swine fever virus, ASFV, proteomics, proteotranscriptomics, macrophages, pathogenicity, immune evasion, MAPK signaling, transcription start site

## Abstract

Since the introduction of a highly pathogenic genotype II isolate of the African swine fever virus (ASFV) into Georgia in 2007, African swine fever (ASF) has gone panzootic. Outbreaks have been reported in Europe, Asia and, more recently, Latin America. Thus, ASFV has become a major threat to the pig industry worldwide, as broadly applicable vaccines are not available. While the majority of ASFV strains show high virulence in domestic pigs and wild boar, variations within the ASFV genome have resulted in the emergence of attenuated strains with low or moderate virulence. However, the molecular basis of the differences in virulence has not yet been discovered. To reveal virulence-associated protein expression patterns, we analysed the proteomes of the natural target cells of ASFV, primary porcine macrophages, after infection with two genotype II ASFV strains displaying high (Armenia 2008) and moderate (Estonia 2014) virulence using quantitative mass spectrometry. Very similar expression patterns were observed for the viral genes, and any differences were limited to the deletions within the Estonia 2014 genome. In addition to the canonical ASFV proteins, twelve novel protein products from recently described transcripts were confirmed in both isolates. Pathway analyses showed that both isolates evoked a similar host proteome response, despite their difference in virulence. However, subtle differences in the manipulation of the proteins involved in the proinflammatory response mediated by the MAPK14/p38 signalling cascade were observed.

## 1. Introduction

African swine fever (ASF) is a disease in swine caused by the ASF virus (ASFV, family *Asfarviridae*). While the course of the infection in the natural hosts (warthogs and bush pigs) is usually mild or inapparent, other members of the *Suidae* family like domestic pigs and European wild boar (*S.scrofa*) develop severe hemorrhagic disease characterised by high fever, apathy, vomiting, diarrhoea, dyspnea, and haemorrhages. In these animals, lethality can reach 100%, depending on the viral strain [1].

Macrophages are the primary target cells of ASFV. Activation of macrophages in response to ASFV infection elicits a strong increase of proinflammatory cytokines, resulting in a so-called cytokine storm [2,3,4]. The multifaceted host defence evasion strategies of ASFV were recently reviewed by Dixon et al. [2]. Targets of ASFV infection include the interferon response, the inflammatory response, apoptosis, and the unfolded protein response. ASFV also impairs autophagy, a cellular process important for maintaining cellular homeostasis and mediating immune responses [5]. 

After entry via clathrin-mediated endocytosis or macropinocytosis, ASFV follows the endolysosomal pathway before viral replication in so-called virus factories in the perinuclear region (reviewed in [6]). Complex interactions with the endoplasmic reticulum (ER) during the ASFV life cycle are observed like an ER-membrane reorganisation in the course of the formation of the inner envelope of the viral particle and the collapse of ER cisternae. Consequentially, the induction of ER stress has been reported [2,7]. Additionally, ASFV impairs autophagy, a cellular process important for maintaining cellular homeostasis and mediating immune responses [5]. 

The large dsDNA genomes of ASFV isolates encode over 150 proteins. Based on the time point of expression within the viral replication cycle, kinetic classes have been defined, with early genes being expressed before, and late genes after, viral DNA replication [8]. However, the expression of early genes can persist in the late phase of infection [9,10,11,12].

In addition to the already large number of known ASFV open reading frames (ORFs), RNA-sequencing and mapping of transcription start sites resulted in the prediction of potential new ASFV-encoded ORFs within the genomes of ASFV strains BA71V and Georgia 2007/1. In total, an additional 203 putative ORFs were predicted for ASFV in recent years [12,13,14]. For some of the ORFs predicted by Chapman and colleagues, experimental evidence for the expression of the corresponding RNA or proteins has been found [12,15,16,17].

The number of genes present in a specific ASFV isolate varies strongly. Variability is especially high for members of multigene families (MGF), which are predominantly located at terminal regions of the viral genome [18]. Genomic deletions can lead to the emergence of naturally attenuated viral strains, which have, for example, been isolated in Portugal (1988–1993; [19]), Latvia (2017; [20]), Estonia (2014; [21]), and China (2020; [22] The strains from Estonia, Latvia, and China descended from the highly pathogenic genotype II Georgia/07 isolate, which was introduced into Europe in 2007 [20,21,22]. In the Estonia 2014 isolate, a deletion of approximately 15 kbp at the 5′-end of the genome resulted in the loss of 27 genes, in comparison with the parental Georgia 2007/1 strain [21]. The lost genes included all MGF 110 members, three members of MGF 360, the p22 homologue KP177R [23], the IL1ß-inhibitor L83L [24], and the uncharacterised ORF L60L. The in vivo host responses to infection with ASFV strains with different pathogenicity were demonstrated to be similar in studies of the transcriptomes [15] or T cell responses [25,26]. However, in vitro experiments showed that the expression patterns of cytokines (for example IL1ß, IL6, and IL12) and the activation of immune response-related signalling pathways (for example cGAS-STING-IRF3 cascade) could vary with the virulence of the isolate [27,28,29,30,31,32,33]. Other pathways, such as the JAK/STAT signalling, are equally targeted by different ASFV isolates to prevent the expression of interferon-stimulated genes [34]. 

One published proteome study compared the responses to infection with a virulent (E75) and an attenuated (E75CV1) homolog ASFV strain observed in porcine lymph nodes [35], which used a combination of two-dimensional gel electrophoresis and matrix-assisted laser desorption/ionization time-of-flight (MALDI-TOF) mass spectrometry (MS) as the analytical platform. While many host pathways were affected by either virus, differential reactions were observed; for instance, a larger number of proteins involved in inflammatory and immunological pathways were differentially expressed after infection with E75CV1, in comparison to E75.

Herein, we analysed the proteomes of primary monocyte-derived macrophages (moMΦ) after infection with two closely related ASFV-genotype II isolates, the highly pathogenic Armenia 2008 [36] and the moderately pathogenic Estonia 2014 [21], using reversed-phase high-performance liquid chromatography (HPLC) coupled to a high-resolution trapped ion mobility spectrometry time-of-flight (timsTOF) mass spectrometer. Macrophages were prepared and infected according to a recently established protocol [37]. One main goal of this study was the characterisation of the expression patterns of viral proteins in the natural target cell and their dependence on the virulence of the respective ASFV strain. To capture the entire possible viral proteome, we also implemented proteotranscriptomic and proteogenomic approaches by adding the translations of recently identified novel ORFs [12] as well as 6-frame translations of the viral genomes to the canonical sequences during the database search of the mass-spectrometric workflow. The second main focus of the study was to compare the macrophages’ responses to infection in order to identify cellular pathways linked to virulence.

## 2. Materials and Methods

### 2.1. Isolation of Primary Porcine Monocyte-Derived Macrophages

Monocyte-derived macrophages (moMΦ) were isolated, selected, differentiated, and infected with ASFV, as described previously [37].

Briefly, blood was drawn from 6–12-month-old female domestic pigs kept at the animal facility of the Friedrich-Loeffler-Institut under the permission LALLF-Nr. 7221.3-2-041/17. Peripheral blood monocytic cells (PBMCs) were isolated using Pancoll animal gradient (density 1.077 g/mL; PanBiotech) and selected for CD172a+ cells using an α-SWC3 specific monoclonal antibody (clone 74-22-15, kindly provided by Dr. U. Blohm, FLI) together with BD IMag magnetic beads (BD Biosciences) as recommended by the manufacturer. Sorted cells were seeded in Primaria cell culture plates (Corning) in PC-1 medium (Lonza), supplemented with 1% (*v*/*v*) penicillin/streptomycin solution (ThermoFisher Scientific) for proteome analysis, or in Iscove′s modified Dulbecco’s medium mixed with Ham’s F-12 nutrient mix (1:1; *v*/*v*), supplemented with 10% FBS and 1% penicillin/streptomycin solution for analysis of MAPK14/p38 expression, and incubated at 37 °C, 2.5% CO_2_. One day after isolation, the medium was replaced by fresh medium supplemented with 5 ng/mL GM-CSF (KingFisher) to enhance differentiation.

### 2.2. Viruses

Infection experiments with ASFV strains Armenia 2008 [36] and Estonia 2014 [21] were carried out in a biocontainment facility that fulfilled the safety requirements for ASF laboratories and animal facilities. Virus stocks were passaged three (Armenia 2008) or four (Estonia 2014) times on PBMCs and titers were determined as tissue culture infectious dose TCID_50_/mL [38] on porcine PBMCs, based on the detection of ASFV-capsid protein p72 by indirect immunofluorescence microscopy.

### 2.3. Infection of moMΦ with ASFV

One day after differentiation, moMΦ were infected with Armenia 2008 or Estonia 2014 with a multiplicity of infection (MOI) of 1. To improve infection rates, cells were centrifugated during the inoculation for 60 min at 600 g and 37 °C [39]. After removal of the inoculum, moMΦ were washed three times with phosphate-buffered saline (PBS), overlaid with fresh medium, and incubated at 37 °C, 2.5% CO_2_. For the preparation of whole cell lysates, moMΦ grown in 6-well culture plates (Corning) were detached with 4 °C cold 50 mM EDTA in PBS, then centrifuged at 4 °C and 250 g for 5 min. The resulting cell pellet was resuspended in 250 µL lysis buffer (2% sodium dodecyl sulfate [SDS] in 0.1 M Tris pH 8.0). After incubation at 95 °C for 10 min, the samples were cooled to room temperature and clarified by centrifugation (14,000× *g*, 10 min), after which the supernatants were collected as lysate. Protein concentrations in lysates were determined by bicinchoninic acid assay [40].

### 2.4. SDS-Polyacrylamide Gel Electrophoresis and Immunoblot Analysis

Equal protein amounts of lysates were separated by SDS polyacrylamide gel electrophoresis (SDS-PAGE) [41] using gradient gels (4–20%, BioRad Laboratories), followed by Coomassie Brilliant Blue-G staining [42] or immunoblot analysis [43]. ASFV proteins p30 and p72 were detected with monospecific rabbit antisera [39,44], kindly provided by Dr. W. Fuchs (FLI). MAPK14 (p38) and its phosphorylated form were detected in immunoblots with commercially available antibodies (Cell Signaling Technology, Germany, #9212, #4511) in combination with a peroxidase-conjugated secondary antibody.

### 2.5. Experimental Design of Proteome Analyses

moMΦ were isolated from two pigs and 5 × 10^6^ cells seeded per 6-well dish. Per pig, triplicate cell cultures were infected with Armenia 2008 or Estonia 2014 at an MOI of 1, and lysates were prepared 6 and 24 h post-infection (hpi). In parallel, mock-infected moMΦ, isolated from one pig, were cultured. Resulting sample groups used for statistical analysis were mock-infected: (*n* = 3), Armenia 2008 6 hpi (*n* = 6), Estonia 2014 6 hpi (*n* = 6), Armenia 2008 24 hpi (*n* = 6) and Estonia 2014 24 hpi (*n* = 6).

The expression of ASFV proteins was compared across time (6 vs. 24 hpi) and across the isolates (Armenia 2008 vs. Estonia 2014). As for the viral proteins, expression levels of the host genes were compared along the time axis for both viruses against controls of mock-infected cells.

### 2.6. LC-MS Analysis of Whole Cell Lysates

All reagents that were used to prepare MS samples were of MS-grade quality. For MS analysis, proteins in lysates were reduced by the addition of DTT to a final concentration of 0.5%, incubated at 95 °C for 10 min, and digested using the filter-aided sample preparation (FASP) protocol [45] with Vivacon 500 ultrafiltration units (MWCO 10 kDa, Sartorius). Trypsin (Promega, Germany, #V5111) was added at a substrate to enzyme ratio of 50:1. Resulting peptides were desalted with Pierce C18 tips (ThermoScientific) following the manufacturer’s recommendations, dried by vacuum centrifugation, dissolved in 0.1% formic acid (FA), and diluted to 0.2 mg/mL for analysis on a nanoElute/timsTOF Pro (Bruker, Bremen, Germany) MS platform.

Per sample, 400 ng of peptides were separated on a nanoElute HPLC (Bruker, Bremen, Germany) equipped with an Aurora (Ionopticks, Fitzroy, Australia) column (25 cm × 75 µm ID, 1.6 µm C18) at a temperature of 40 °C with a flow rate of 400 nL/min. Solvent A was 0.1% formic acid (FA) and solvent B was 0.1% FA in acetonitrile. Peptides were eluted with a 115 min binary gradient from 2% to 16% solvent B (0–60 min), 15–24% solvent B (60–90 min), 24–34% solvent B (90–105 min), 35–95% solvent B (105–107 min), and 95% solvent B (107–115 min).

The timsTOF Pro instrument was equipped with a CaptiveSpray nano electrospray ion source (Bruker) and was operated in Parallel Accumulation and Serial Fragmentation (PASEF) mode using the standard data-dependent acquisition (DDA) method for proteome analysis (1.1 sec cycle time) recommended by the manufacturer.

### 2.7. Proteome Analysis

For the identification of proteins, raw MS data were processed with MaxQuant version 1.6.17.0 [46,47] using sequence databases compiled from the host Sus scrofa (downloaded from Ensembl repository [48], and the sequences of the ASFV isolates Georgia 2007/1 (GenBank FR684268.2) and Estonia 2014 (GenBank LS478113). For the identification of novel ORFs (nORFs), the databases were complemented with sequences provided in [12].

The results from Maxquant were further processed using the statistical programming language R [49] and analysed with Perseus v1.6.15.0 [50].

Host gene annotations from the Kyoto Encyclopedia of Genes and Genomes (KEGG) repository [51] and the Gene Ontology (GO) database [52] were processed with in-house R scripts for use in Perseus software. If annotations to the porcine genes were unavailable, annotations to the human orthologs of the identified porcine genes were used after cross-referencing with the R-package gprofiler2 version 0.2.1 [53]. Annotations to viral genes were added from an in-house database based on literature research.

Statistical analysis was performed in Perseus v1.6.15.0 based on log10-transformed label-free quantification (LFQ) values using the workflow shown in Figure S1. Pairwise comparisons between groups were performed in Perseus using left- and right-sided Student’s T-tests with an FDR of 0.05.

For host gene expression analysis, porcine protein identifiers were referenced to the corresponding HUGO Gene Nomenclature Committee (HGNC) [54] gene symbol using the R-package gprofiler2 version 0.2.1 [53]. Lists of differentially-expressed genes (DEG) (infected vs. mock) used for the term enrichment analysis are provided in Supplementary File Table S1C.

For enrichment and cluster analysis of biological process GO-Terms (GO:BP) and KEGG pathways, the R-package gprofiler2 and Cytoscape version 3.9.1 [55], together with the packages ClueGO version 2.5.8 [56] and CluePedia version 1.5.8 [57], was used. Figures were generated using Biorender.com, the R-package ggplot2 [58] accessed at https://cran.r-project.org/package=ggplot2, accessed on 10 August 2022, Perseus v1.6.15.0, and Cytoscape version 3.9.1.

### 2.8. Role of p38 during ASFV Infection

To assess the impact of activation or inhibition of p38 on ASFV replication, 1.5 × 10^6^ moMΦ per sample were infected with an MOI of 1 either in the presence of conditioned medium or the p38-inhibitor BIRB796 (25 nM in DMSO, Tocris), or after a 4 h pretreatment with the p38-activator TNFα (20 ng/mL in 5% trehalose, Biolegend). The conditioned medium was prepared by three sequential filtrations of the virus stock through 0.1 µm sterile syringe filters (qpore). At 6 and 24 hpi moMΦ lysates were prepared and the expression and phosphorylation of MAPK14/p38 was assessed by immunoblotting. Supernatants for the determination of virus titers were collected at 24 hpi.

### 2.9. Cell Viability

For assessment of cell viability after treatment with TNFα and BIRB796, 2.5 × 10^5^ of the CD172a^+^ monocytes, isolated and differentiated as described above, were seeded per well and treated with 20 ng/mL TNFα or 25 nM BIRB796 or a combination of both for 6 or 24 h. After the incubation period, cell viability was tested with PrestoBlue™ HS Reagent (ThermoFisher, Germany). Based on fluorescence (excitation 560 nm, emission 590 nm) the percentage of viable cells in treated wells was calculated and given as the ratio to the mean viability of naïve moMΦ.

### 2.10. Modelling of Protein Structures

For structural modelling and functional prediction of the N-truncation variants of pK78R (Uniprot ID: Q89769), pB646L (PDB: 6KU9), and p150 (Uniprot ID: A0A3S9JJG0) the following bioinformatic tools and databases were used: Alphafold [59], IUPred2A [60], DISOPRED [61], InterPro [62], Phobius [63], PSIPRED [64,65], and TMHMM [66] for prediction of transmembrane domains. Structure predictions using AlphaFold used ‘AlphaFold Colab’ available via Google Colab, with a Colab Pro account (https://colab.research.google.com/github/deepmind/alphafold/blob/main/notebooks/AlphaFold.ipynb (accessed on 3 March 2022)). This Google Colab notebook used a simpler version of AlphaFold v2.1.0. with a subset of the Big Fantastic Database (BFD). For pK78R, the ORF sequence was input into AlphaFold Colab, the runtime was connected and hardware accelerator set as GPU, before Run All was selected. The output consisted of three graphs. First, a summary of the multiple sequence alignment of templates found in the BFD, representing the “number of sequences per position”, second, graphs of “Predicted Local Distance Difference Test (pLDDT) per position” (a representation of confidence across the sequence), and third, the “Predicted Alignment Error”, indicating overall model quality. These graphs are found in Figure S12A–C, respectively. The model was viewable within the Colab notebook with the residues shaded according to pLDDT score (Figure S12D), and then downloaded in PDB format. All models and PDB structure figures were generated using UCSF Chimera [67].

### 2.11. Calculation of Genome Coverage 

ORFs from the Georgia 2007/1 genome (GenBank FR682468.1), were taken from the new transcriptomic annotations. This included the genome coordinates for the canonical ORFs and the novel ORFs (nORFs) with transcription start sites (TSSs) detectable via cap-analysis gene expression [12]. The nORFs with intra-ORF TSSs were excluded from genome coverage calculations as they were already found within ORFs. To calculate the genome coverage from ORFs, BEDTools [68] software was used. Firstly, a gene feature file (GFF) was made from the updated genome annotations. Any overlapping genome coordinates were merged in the GFF, using the mergeBed function. The length in bp of each ORF was then calculated, totalled per strand, and divided by the total genome length of 189,344 bp. The percentage coverage was therefore the mean of coverage across both the plus and minus strands.

### 2.12. Data Availability

The mass spectrometry proteomics data have been deposited to the ProteomeXchange Consortium (http://proteomecentral.proteomexchange.org (accessed on 8 August 2022)) via the PRIDE partner repository [69] with the dataset identifier PXD036402 and DOI 10.6019/PXD036402.

## 3. Results

**Figure 1:**
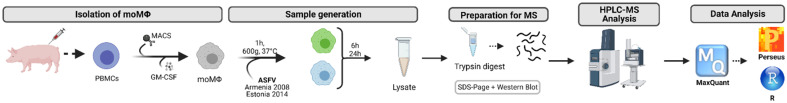
Representation of the proteomic workflow. Peripheral blood monocytic cells (PBMCs) are isolated, selected for CD172a+ monocytes, differentiated into monocyte-derived macrophages (moMΦ), and infected with Armenia 2008 or Estonia 2014. Proteins are extracted at 6 and 24 hpi, digested with trypsin and analysed on a timsTOF LC-MS platform. Data analyses are carried out with MaxQuant and Perseus software or in-house R scripts.

In this study, we compared the expression patterns of ASFV genes and the host response of moMΦ infected with two ASFV isolates of high (Armenia 2008) or moderate (Estonia 2014) pathogenicity, following the workflow described in Figure 1. Cell batches were infected with either virus, harvested 6 and 24 hpi, and processed for proteome analysis on a timsTOF Pro MS platform. The sequence database used for protein identification was compiled from the canonical host and virus proteins and a set of 175 hypothetical proteins corresponding to novel ORFs that were recently identified under similar experimental conditions [12].

Before MS-analysis, sample homogeneity and ASFV infection were confirmed by SDS-PAGE and immunoblot analysis against early and late viral proteins p30 and p72, respectively (Figure S2).

### 3.1. Expression of Viral Proteins

**Figure 2:**
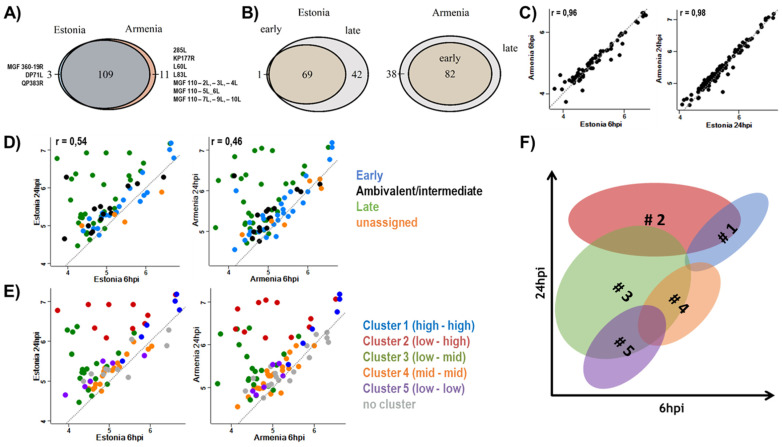
Expression of viral proteins after infection of monocyte-derived macrophages (moMΦ) with African swine fever virus (ASFV) strains Armenia 2008 or Estonia 2014. (**A**) Qualitative comparison of the expression of viral genes in infected moMΦ after infection with Armenia 2008 or Estonia 2014. (**B**) Comparison of ASFV proteins expressed at early (6 hpi) and late (24 hpi) time points after infection with Estonia 2014 (**left**) or Armenia 2008 (**right**). (**C**) Correlation analysis of the expression levels of viral proteins after infection with Armenia 2008 or Estonia 2014 at 6 hpi (**left**) and 24 hpi (**right**), based on log10 LFQ (label-free quantitation) values. (**D**,**E**) Comparison of ASFV protein expression at early (6 hpi, *x*-axes) and late (24 hpi, *y*-axes) stages after infection with Estonia 2014 (**left**) and Armenia 2008 (**right**). Colours represent published kinetic classes (**D**) or recently proposed revised temporal clusters [12] (**E**). (**F**) Schematic summary of temporal expression clusters based on Cackett et al. (2022) [12]. (**C**–**E**): Quantitative data are based on means of log_10_-transformed label-free quantification values representing 6 replicates per condition, r = Pearson’s correlation coefficient, grey dotted line = dissecting line as reference.

In total, proteins corresponding to 123 of the currently annotated ASFV genes could be identified at 6 hpi or 24 hpi, 120 of the 189 Armenia 2008 ORFs, and 112 of the 163 Estonia 2014 ORFS. Detailed MS data are given in Supplementary File Table S1A. Of these, 3 and 11 were exclusively expressed after infection with Estonia 2014 or Armenia 2008, respectively (Figure 2A). As the panel of ASFV proteins exclusively detected in Armenia 2008 infected cultures was coherent with their absence from the Estonia 2014 genome, due to the deletion at the 5′-end, the expression patterns of the remaining viral proteins in moMΦ were very similar between both strains. Likely, the panels of proteins that were expressed after 24 hpi in addition to those present at the earlier time point (Figure 2B) were similar for both strains (38 for Armenia 2008, 42 for Estonia 2014).

Quantitative comparison of ASFV-protein expression levels 6 and 24 h after infection with Armenia 2008 and Estonia 2014 (Figure 2C) showed a strong correlation between the expression levels of viral genes from both isolates at both time points (Pearsons’s correlation coefficient 0.96 and 0.98 at 6 and 24 hpi, respectively). 

The protein expression levels at both time points are compared in Figure 2D for both strains separately, with colour coding according to the temporal classification into early, ambivalent/intermediate, late, and unassigned, as found in the literature. Most, but not all, genes with late expression kinetics were expressed stronger at 24 hpi than at 6 hpi. Such exceptions of late genes, which were expressed with constant levels at the early and the late time point, were QP509L and C147L from both strains and QP383R from Estonia 2014. Protein levels of most genes with early kinetics remained stable over time. The levels of the gene products from I9R and ASFV_G_ACD_00600 of both viruses dropped significantly at 24 hpi (Figure S3). Thus, these genes, lacking a temporal classification so far, may have belonged to the early class. Furthermore, gene products of I7L and members of the MGF 110 were detected at 6 hpi and showed no strong accumulation at 24 hpi (Figure S3), suggesting early kinetics. In contrast, the expression of ASFV_G_ACD_01020 was limited to samples harvested 24 hpi with either isolate, indicating preferential expression at late stages of infection.

Figure 2E shows the same data as Figure 2D; however, the colour-coding follows the revised temporal classification, based on transcript abundance, which was proposed by Cackett and colleagues [12], shown schematically in Figure 2F. Clustering according to transcript and protein levels at early and late times of infection correlated well for the majority of proteins. However, two exceptions were noted. First, the product of C129R, currently classified as ambivalent, located between cluster#3 (low to mid) and cluster#2 (low to high), rather than clearly with cluster#3, and second, the product of the late gene A137R associated closer with cluster#1 (high-high), rather than with the typical late clusters (#2, #3).

In addition to the expression analysis of ORFs currently annotated in the ASFV genomes, we used a proteotranscriptomic approach to explore the expression of novel ORFs (nORF) which were recently annotated within the Georgia07 genome based on mapping of transcription start sites [12]. Indeed, 364 spectra could be mapped to peptides from 65 nORFs (Supplementary File Table S1B). Of these peptides, 19 were unique and confirmed the expression of eleven nORFs. Six of them could be confirmed even though they were annotated within an existing ORF (K205R, DP238L, CP204L, B646L, CP2475L, and K78R), as the presence of the N-termini of the nORF was substantiated by mapping of the respective peptides—which had been identified by MS. Five of the nORFs confirmed by MS map to genome regions were currently not annotated at all (Table 1, Figure S4). For nORF identifications with only one unique peptide, the annotated MS spectra are provided in Figure S5. Acetylation of the N-terminal peptide could be detected for 717 host proteins and 27 ASFV proteins, including nORF_176208. The expression of additional nORFs representing other truncated variants of annotated ASFV-ORFs could not be confirmed, as the N-terminal peptides of the novel ORFs could not be identified (Supplementary File Table S1B). 

Based on the time point of detection of the unique peptides and comparison of their expression levels between 6 and 24 hpi, we proposed 4 nORFs as early genes, 2 as ambivalent, and 5 as late (Table 1).

In addition to the proteotranscriptomic approach, we also matched the MS data to a database that was constructed from 6-frame translations of the genome in order to identify ORFs that may have escaped annotation in the genome, as well as using TSS mapping. A cluster of two peptides was detected that mapped to a potential reverse reading frame located at the C-terminal region of the F778R gene. Reevaluation of the published TSS mapping data [12] allowed mapping of both peptides to a TSS at 59,454 (minus strand). This could generate two isoforms, arising from alternative start codons, at positions 59,430 or 59,421 in the ASFV-Georgia genome, encoding for proteins of 49 or 46 aa, respectively (Figure S6). Due to the location of the identified peptides at the shared C-terminus of both possible isoforms, MS data were only able to confirm the presence of a corresponding protein, but not which of the isoforms was translated. Both transcriptome and proteome data indicated expression at the late stage of infection.

Of the confirmed nORFs, four coded for truncated variants of structural proteins, including the virion-forming proteins p72 (pB646L), the pp220-derived p150 (pCP2475L), and p10 (pK78R). To evaluate the possible effects of the truncation on capsid and core formation or DNA-binding by p10, we compared the structures of the full-length and truncated variants using in silico structure predictions.

**Figure 3:**
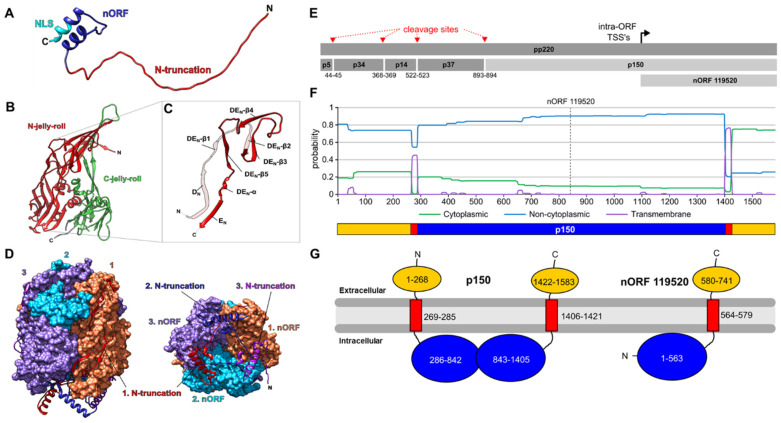
(**A**) AlphaFold structure predictions of full-length p10 (K78R). The 37-residue region lacking in the N-truncated variant is shown in red, and the 41-aa potential nORF_63974 protein is in blue. The C-terminal sequence KKIKRSK in cyan is predicted to contain a nuclear localization signal (NLS). (**B**–**D**) The structure of p72 PDB: 6KU9, and the structure prediction of nORF_105178. (**B**) The structure of a p72 monomer with the N and C-terminal jelly-roll domains, coloured and labelled in red and green, respectively. (**C**) The structure of the p72 DE_N_-loop, with secondary structure features annotated. The region missing in the N-truncated variant nORF_105178 (transparent) consists of D_N_, DE_N_-β1, and DE_N_-β2. (**D**) Side (left) and bottom (rotated by 270°, right) view of the p72-homotrimer, with surface representations of monomers 1, 2, and 3 in salmon, cyan, and lilac, respectively. The residues missing in nORF_105178 are shown as a ribbon. (**E**–**G**) The N-truncation of p150 and its potential effect on protein function. (**E**) Schematic representation of pp220 processing by protease pS273R into proteins p5, p34, p14, p37, and p150 (red arrows). The black arrow represents the intra-ORF TSS within the p150 sequence giving rise to nORF_119520. (**F**) Probability plot predicting transmembrane (TM) and cytoplasmic domains of p150 using the Phobius web server [63]. (**G**) Schematic representation of full-length p150 and nORF_119520 domains predicted by PSIPRED and TMHMM in relation to a membrane (grey). The numbered residues are coloured according to the predicted location by the PSIPRED: MEMSAT-SVM algorithm; extracellular location is yellow, the transmembrane helices are red, and intracellular location is blue.

Currently, no structure of ASFV DNA-binding protein p10 is available. Therefore, to investigate the effect of an N-truncation, we predicted its structure using AlphaFold [59] (Figure 3A). This model showed a long-disordered region, followed by a structured helix-turn-helix-like domain at the C-terminus. It was supported by sequence predictions using IUPred2A [60], DISOPRED [61], and InterPro [62], suggesting a disordered region residing from position 1 to ~30–40 of the full-length p10. This was particularly interesting because nORF_63974, which we showed to be both transcribed and translated, would omit residues 1–37, including this disordered region (red in Figure 3A). The full-length p10 protein was expressed recombinantly in E. coli, showing single and double-stranded DNA binding capacity [70]. Deletion variants generated by Nunes-Correia et al., (2008) demonstrated the necessity of only the C-terminal region for localization into the nucleus, especially residues which contain a nuclear localization (NLS)-like sequence KKIKRSK (cyan in Figure 3A). Therefore, we predicted the p10 N-truncated variant nORF_63974 would retain nuclear localization. However, we we could not predict if its DNA-binding capacity would remain, as it is not known whether DNA binds to the N- or C-terminal regions in full-length p10. 

It was interesting to observe N-truncation variants arising from the B646L gene, encoding the p72 capsid protein which is essential for virion assembly. There are now structures available of p72 alone [71] (Figure 3B), as well as in the context of the ASFV capsid structure [72,73] enabling a structure-based analysis of N-truncated p72 proteins generated by intra-ORF TSS’s like nORF_105178. This nORF began 127 residues after the p72 start codon, which, according to the structure from Liu et al., 2019 would omit the first 5 β-strands. This truncation would not form β-strands D_N_, DE_N_-β1, and D_EN_-β2, required to form the ‘DE_N_-loop’ (Figure 3B), which was important for the p72 homotrimer formation [74]. The structural disruption of nORF_105178 was clearer when viewing the p72 homotrimer (Figure 3C), which demonstrated where these truncated residues were located along the interface of p72 monomers, as well as their intertwining underneath the double jelly-roll base (Figure 3D). This all suggested that nORF_105178 proteins could not form a stable p72-like homotrimer.

Understanding the impact of N-truncations on the p150 protein was more complex than for p10 and p72, due to its greater size and our minimal understanding of its function. Additionally, full-length p150 is formed from proteolytic cleavage of the polyprotein pp220 (encoded by gene CP2475L) by cysteine protease pS273R, which also generates proteins p5, p34, p14, and p37 (Figure 3E). The novel intra-ORFs of CP2475L (nORF 118699 and nORF_119520) were particularly interesting, since both were located within the p150 sequence and downstream of the final proteolytic cleavage site (Figure 3E). Therefore, their protein products would be synthesised independently of the proteolytic activity of pS273R.

We detected peptides arising from nORF_119520 and, despite it being the longest nORF within the p150 sequence at 741 residues (nORF_118699 encodes 471), it only encompassed roughly half of the p150 sequence. We would have expected this to greatly affect function, but current understanding of the functional domains and structure of p150 is extremely poor due to the lack of characterised sequence homologs. However, multiple sequence prediction tools have suggested that p150 contains two putative transmembrane (TM) domains. These separated the cytoplasmic region (the majority of p150) from non-cytoplasmic N- and C-termini (Figure 3F,G and Figure S7). This suggested that full-length p150 was anchored to membranes via two separate TM domains. The truncated variant of p150 generated by nORF_119520 effectively cut the protein in half, retaining the C-terminal TM-domain anchor, but losing a substantial amount of the cytoplasmic region and the N-terminal TM anchor and extracellular domain (Figure 3G).

### 3.2. Host Response to Infection

To evaluate the host response to infection with Armenia 2008 or Estonia 2014, we identified DEG as a basis for GO-term enrichment and pathway analysis. We also analysed the expression profiles of individual genes known to play a role in viral infections. Statistical testing was performed with Perseus software based on the LFQ quantitation of the MS results obtained for 3376 proteins with Maxquant software (Supplementary File Table S1C). Genes that were differentially expressed 6 or 24 hpi were subjected to a term enrichment analysis to identify cellular pathways affected at early and late times of infection. The detailed results are provided in Supplementary File Table S2 and a summary can be found in Table 2. 

We noted a large overlap of the pathways that were affected by infection with either of the two viruses, among them some related to cell death (GO:0008219, GO:0010941, GO:0012501, GO:0006915, GO:0043067, GO:0042981): the phagosome (KEGG:04145), the lysosome (KEGG:04142), and the proteasome (KEGG:03050). However, some responses were strain specific. For Estonia 2014-infected moMΦ, these terms were associated with the immune response and its regulation (GO:0006955, GO:0002376, GO:0045087, GO:0050778, GO:0002684, GO:0050776, GO:0002682), especially antigen processing and presentation (KEGG:04612, GO:0019884, GO:0002478, GO:0002474, GO:0002504) and cytokine response (GO:0071345, GO:0001816, GO:0001817, GO:0034097, GO:0001819, GO:0023056). In contrast, pathways pointing at autophagy-related diseases such as Parkinson’s, Alzheimer’s, or Huntington’s disease (KEGG:05010, KEGG:05014, KEGG:05012, KEGG:05016) and the response to ER-stress (GO:0036503, GO:0030433, GO:0034976) were only enriched after infection with the highly pathogenic Armenia 2008 isolate.

**Figure 4:**
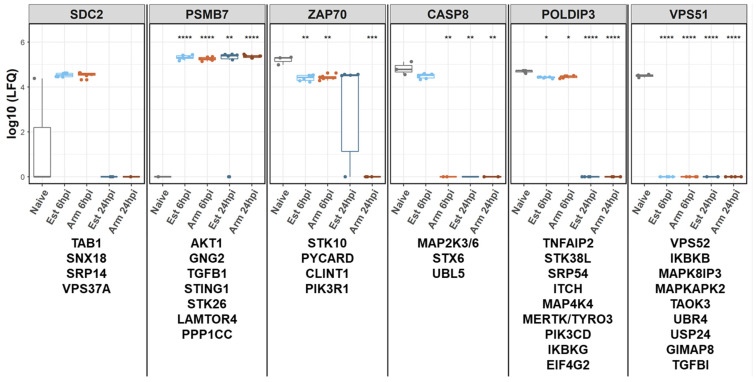
Expression profiles of porcine genes during African swine fever virus (ASFV) infection of monocyte-derived macrophages with Armenia 2008 (Arm) or Estonia 2014 (Est). Captions of the panels indicate one exemplary representative of the groups of host genes listed below the respective graphs sharing specific expression patterns. Asterisks indicate *p*-values of 0.05 (*), 0.01 (**), 0.001 (***), and 0.0001 (****).

As indicated by the results of the enrichment analysis, we expected only smaller numbers of proteins with divergent protein expression levels in the comparison between the proteomes of the infected moMΦ. Therefore, we filtered the expression data for certain quantitative profile patterns in Perseus software to detect smaller protein groups or individual proteins that were affected by infection (Figure 4). Details can be found in Supplementary File Table S1C.

Based on the patterns of the expression profiles in cells infected with either virus and over time, six groups of host genes with altered expression levels after infection with Armenia 2008 or Estonia 2014 were defined (Figure 4). Specifically, these six groups were: (1) genes transiently expressed at 6 hpi after infection with any of the two viruses (example Syndecan 2 (SDC2)), (2) genes expressed in naïve moMΦ but lacking after infection with any of the two viruses at any time point after infection (e.g., Vacuolar protein sorting-associated protein VPS51), (3) genes expressed in naïve moMΦ and Estonia 2014-infected moMΦ at 6 hpi (e.g., caspase 8 (CASP8)), (4) genes expressed at 6 h and 24 h after infection with any of the viruses but not after mock infection (e.g., proteasome subunit beta 7 (PSMB7)), (5) genes not expressed at 24 hpi after infection with any of the two viruses (e.g., polymerase delta-interacting protein POLDIP3), and (6) genes not expressed in Armenia 2008-infected moMΦ at 24 hpi (e.g., tyrosine-protein kinase ZAP70).

In addition to genes noted in the qualitative analysis above, quantitative analysis revealed that several genes were present in the mock-infected cells but significantly downregulated in at least some of the infected samples. These included cathepsins (CTSH, CTSD), cell surface-expressed proteins such as CD14, macrophage scavenger receptor (MSR1) and the macrophage mannose receptor MRC1, integrins (ITGAL, ITGAM, ITGB2), apolipoprotein E (APOE), C-type lectin domain family 4 member CLEC4M, and vacuolar protein sorting-associated protein VPS35. Others, such as interferon-induced GTP-binding protein MX1, importin subunit alpha-5 KPNA1, and members of the HNRNP complex, were upregulated after infection (Supplementary File Table S1C, Figure S8A).

In contrast to these genes with similar regulations after infection with either virus, others showed differential regulations after infection with the two viruses. Among these were the microtubule-actin cross-linking factor 1 (MACF1), GTPase IMAP family member 4 (GIMAP4), sialoadhesin (SIGLEC1), E3 ubiquitin-protein ligase RNF213, and the cation-independent mannose-6-phosphate receptor IGF2R (Figures S8A and S9). 

As there was no obvious functional connection between these genes, we subjected a list of 95 porcine genes (Table S3) to pathway enrichment and network analysis to identify common pathways and potential central regulators. For analysis and visualization of the results, Cytoscape software was used together with the plugins ClueGo and CluePedia. 

Enriched pathways and terms primarily related to the cellular immune response, such as C-type lectin receptor-, TNF-, MAPK-, RIG-1-like and TLR-like signaling pathways, and pathways related to infections with different viruses (EBV, hepatitis B and C, HIV, Influenza A, Measles) (Figure S10, Supplementary File Table S4). Network analysis based on the node degrees revealed RIPK1, MAPK14, TAB1, and NFκB as central nodes (Figure S10B,C).

### 3.3. Analysis of p38 during ASFV Infection

**Figure 5:**
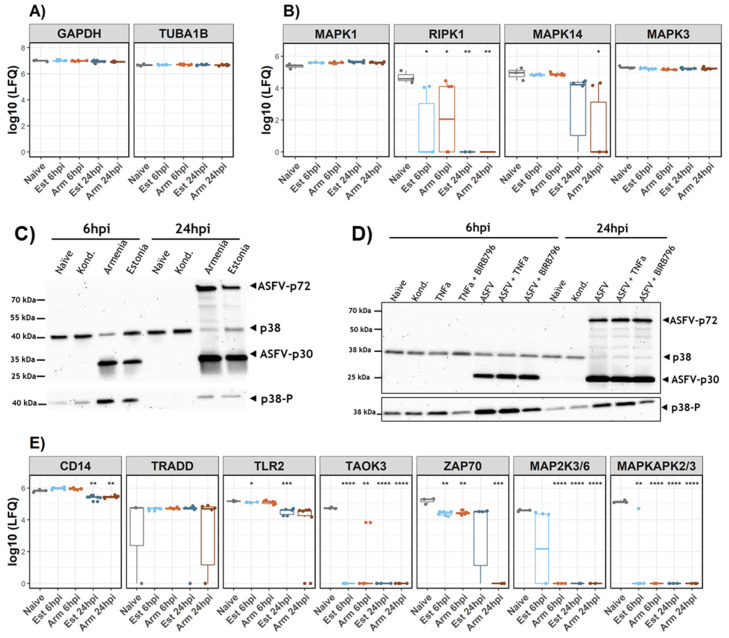
Expression levels of housekeeping genes GAPDH and TUBA1B (**A**) as references for described modulation of MAPK1, MAPK3, and MAPK14 levels (**B**) and genes involved in MAPK14/p38 signaling cascade (**E**) based on label-free quantification of MS data. (**C**) + (**D**) Immunoblot analysis of African swine fever virus (ASFV) gene products ASFV-p30 (early), ASFV-p72 (late), and MAPK14/p38 expression and its phosphorylated form in monocyte-derived macrophages (moMΦ) after infection with Armenia 2008 (Arm) or Estonia 2014 (Est) with an MOI of 1 after 6 and 24 hpi. (**D**) To assess the impact of MAPK14/p38 pre-stimulation or inhibition during infection with Armenia 2008, moMΦ were treated with 20 ng/mL TNFα for 4 h or 25 nM BIRB796 during the infection period, respectively. Asterisks indicate *p*-values of 0.05 (*), 0.01 (**), 0.001 (***), and 0.0001 (****).

Since the effects of ASFV infection on NFκB-mediated signaling events have been studied more extensively (reviewed in [2]), we focused on the genes involved in MAPK signaling, MAPK14 and RIPK1. As there is a high degree of cross-talk between the MAPK signaling pathways, the expression levels of the other major MAPK kinases were included in a reanalysis of the MS data. While no expression of JNK could be detected, expression levels of MAPK1 and MAPK3 were unaffected by ASFV infection. Contrary to that, expression levels of MAPK14 and RIPK1 were significantly reduced at 24h after infection with any of the viruses (Figure 5B). Since it is known that RIPK1 transmits the TNFR-mediated signal via MAPK14 kinases [75], we analysed the expression and phosphorylation of MAPK14/p38a in moMΦ by immunoblotting under different conditions, including ASFV infection, stimulation, and inhibition of the MAPK signaling pathway. To exclude the influence of the inoculum, a control with conditioned medium was added. The results showed that, at 6 hpi, Armenia 2008 and Estonia 2014 both induced phosphorylation of MAPK14/p38 without influencing total protein levels, while at 24 hpi, levels of phosphorylated and total MAPK14/p38 decreased. Treatment with conditioned medium did not influence p38 expression or its phosphorylation at 6 or 24 hpi (Figure 5C).

Activation or inhibition of MAPK14/p38 using TNFα or BIRB did not decrease cell viability significantly. Furthermore, neither of the treatments affected the ASFV protein expression levels or significantly impacted viral titers (Figure 5D and Figure S11).

**Figure 6:**
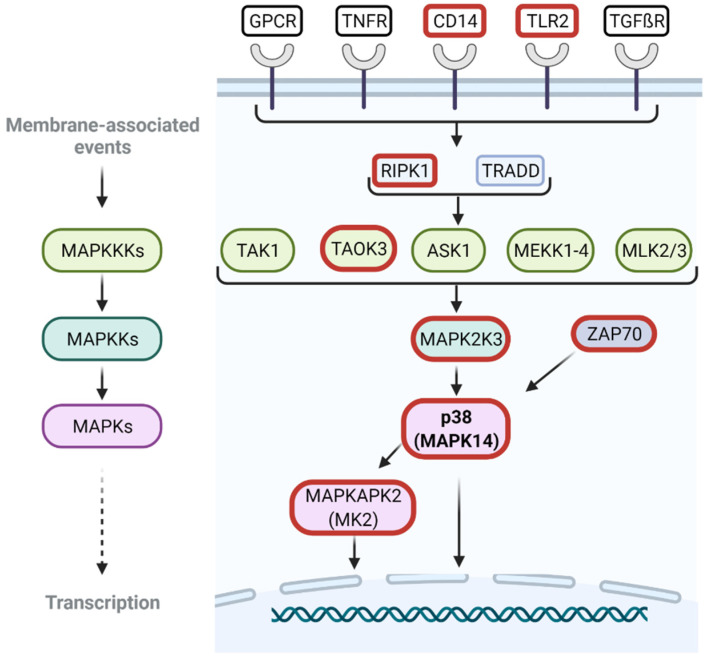
Graphic summary of genes involved in MAPK14/p38a signaling. Genes with decreased expression levels reported in the present study are highlighted by bold red outlines.

Following the confirmation of ASFV-induced modulation of MAPK14/p38 activation and expression, the expression levels of other genes also involved in MAPK signaling cascades were assessed in the MS data. While other kinases involved in ERK signaling were unaffected (Figure S8B), genes involved in MAPK14/p38 signaling, including TAOK3, MAP2K3/6, and MAPKAPK2/3, were downregulated compared to naïve moMΦ in response to the ASFV infection (Figure 5E and Figure 6). Interestingly, differences between Armenia 2008- and Estonia 2014-infected moMΦ were noted for MAP2K3/6 and ZAP70 expression. While MAP2K3 could still be detected at 6 hpi in Estonia 2014-infected samples, no expression was observed in MS data of ASFV Armenia 2008-infected moMΦ at the same time point. Reduced levels of ZAP70 were observed at 6 hpi for both viruses, which were maintained until 24 hpi after infection with Estonia 2014, but dropped below detection levels in Armenia 2008-infected moMΦ at the later time point.

## 4. Discussion

Despite many efforts, the molecular basis of the pathogenicity of ASFV is not well understood. The large size of the ASFV genome may allow an intricate manipulation of the host cell and evoke multifaceted mechanisms to modulate the host immune response. The resulting dysregulations remain obscure in many aspects (reviewed in [2]). 

The current study aimed to increase the understanding of ASFV pathogenicity by comparison of the proteomes of porcine moMΦ, the primary target cell of ASFV, after infection with two differentially virulent ASFV strains that were well characterised in vivo. To separate potential differences in the early and late phases of infection, the analyses were performed at 6 hpi and 24 hpi. The application of quantitative high-resolution mass spectrometry allowed for quantitation of the expression of the majority of all ASFV genes in a time-dependent manner. Quantitative modulations of the host protein levels were used to identify differentially-expressed proteins and cellular pathways manipulated by the viruses using biostatistical analysis. In addition, the expression profiles of the host proteins were screened for striking temporal patterns to identify individual proteins with a potential role in ASFV infection in general and concerning differences in virulence.

The expressions of the viral proteins in infected moMΦ were very similar, irrespective of the virulence of the two isolates. Qualitative differences reflected the loss of 26 genes by a large deletion in the Estonia 2014 genome [21]. Expression levels of the genes present in both isolates showed a strong correlation. These observations correlated well with the RNA levels after infection of macrophages and Vero cells with the highly pathogenic Georgia07 and the attenuated BA71V strain, respectively [12,14]. Additionally, the quantitative temporal expression patterns of ASFV genes suggested by Cackett et al., 2022 [12] on basis of mRNA analysis, could be confirmed (Figure 2)—and could apply to the expression of viral proteins by both isolates as well. Although corresponding proteins were found for the majority of annotated ORFs, some remained undetected, indicating either lack of expression in moMΦ or expression levels below the detection limits of MS.

Structural protein pA137R clustered with early genes, rather than late genes as expected [76,77]. For three viral proteins—pI7L, pASFV_G_ACD_00600, and pASFV_G_ACD_01020—the proposed early temporal expression kinetics based on transcript analysis [12] were confirmed at the protein level. Similarly, the early classification of members of MGF 110, based on their detection at 6 hpi without a strong accumulation at 24 hpi, was confirmed [14]. 

While viral mRNA and protein levels seemed to correlate well, this is not generally the case for host proteins [78]. Examples of differential regulations of transcript levels, taken from the literature [12,15], and protein levels, observed in the present study, include SIGLEC1, CD163, and ARG1. Furthermore, different modulations of individual host cell transcript levels have been observed after infection with specific ASFV isolates, indicating a notable influence of experimental conditions [3,12,15,79].

It is important to note that the current analysis was based only on the measurement of static protein levels. Thus, proteins synthesised in the early phase of infection which did not degrade below detection limits would have also been detected in the late phase. Moreover, in two early 2D-electrophoretic studies based on the incorporation of radioactive precursors [10,11], it was shown that the synthesis of early-expressed proteins can persist in the late phase of infection. Understanding the metabolism of all ASFV proteins, including synthesis and decay rates, will require a different mass spectrometric approach.

Another step towards understanding the replication of ASFV may be the identification and functional characterization of alternative transcripts and their corresponding proteins. In a proteotranscriptomic approach, we included a set of recently characterised alternative transcripts into the sequence database used for the MS analysis and could confirm the existence of the corresponding proteins of eleven novel ASFV-ORFs. For six of them, the N-terminal peptides corresponding to alternative translation starts were identified. This indicated that ASFV generally used alternative translation starts, and not only as compensation for mutations affecting start codons [13]. In this way, we confirmed proteins corresponding to alternative ORFs encoding parts of the capsid protein p72 (pB646L) or DNA-binding protein p10 (pK78R) [70]. For two ASFV proteins and their respective N-truncated variants, we predicted structural models to assess potential functions. 

The full-length p10 (encoded by K78R) accumulates within the nucleus during the late stage of infection [80], which may also be the case for the truncated protein, as the nuclear location signal is retained in the corresponding nORF_63974. However, an artificially truncated variant based on the sequence of genotype I strain L60 which lacked the first 30aa or 53aaa localised to the cytoplasm, not the nucleus [80]. Structural modelling identified the lack of the N-terminal disordered region as the main difference between full-length pK78R and nORF_63974. Disordered protein regions are often involved in protein interactions and signalling events [81,82]. The full-length product of K78R has been shown to bind DNA [70]; however, a DNA-binding domain has not yet been mapped. Hence, the possibility remains that the DNA-binding domain of pK78R is located within the disordered region. Consequently, the extent to which the truncation by 37aa affects the subcellular localization needs to be evaluated, and the DNA-binding capacity of nORF_63974, as compared to pK78R, requires more detailed investigation.

The detection of a protein corresponding to nORF_105178 confirmed the expression of at least one truncated variant of the major capsid protein ASFV-p72 encoded by the B646L gene. The commonly observed additional bands in ASFV-p72-specific immunoblots between ~70 and 40 kDa (Figure 5C,D) could represent truncated variants like nORF_104896 and other (currently not identified) alternative p72-derived proteins. The truncation of the pB646L N-terminus by 127aa in nORF_105178 impaired the formation of the ‘DEN loop’ which is important for homotrimer formation [71]. Furthermore, the N-terminal residues of p72 are important for capsid assembly [72]. Therefore, the ability of the truncation variants to form homotrimers might have been impaired, as the sequence was located near the interface of the interacting p72 proteins [71]. Whether the presence of truncated p72 variants interfered with virus replication or morphogenesis—for instance, by inhibiting the integration of full-length p72 into functional capsids—remains to be determined. 

ASFV polyprotein pp220 (CP2475L) is the precursor of the mature proteins p150, p5, p34, p37, and p14, which result from proteolytic cleavage of pp220 by the viral protease pS273R [83,84]. In particular, p150 results from the cleavage at the most C-terminal cleavage site of pp220, indicating that expression of nORF_119520 from a TSS downstream would be independent of pS273R activity. For the correct formation of the viral particle, membrane association of p150 is required; however, this has only been observed for correctly processed p150 association with the membrane [85]. Sequence-based modelling of p150 and the corresponding novel ORF predicted the presence of two membrane-associated domains within p150, connected by an intracellular domain. The truncation consists roughly of the C-terminal half of p150, retaining the C-terminal TM domain and a part of the intracellular sequence. However, variations in the predictions of different modelling algorithms regarding the N-terminal TM domain (p150 aa 269–285) leave open the possibility of a cytoplasmatic localization of the N-terminal domain of p150, if the TM is peripherally attached to the membrane but not spanning it completely. Amphipathic helices acting as membrane anchors have been described for mature products of flaviviral polyproteins of HCV [86] and pestiviruses [87] or the poliovirus 2C protein [88].

Due to the large size of the truncation and the impact it has on protein topology we expected the function of nORF_119520 to be markedly different from the full-length p150. It seemed questionable whether the products of nORF_105178 and nORF_119520 could participate in the formation of the virion. However, our current understanding of the functional domains and structure of p150 remains too poor to formulate any specific hypotheses in this respect. 

The protein repertoire of ASFV may be larger than expected and exceed the 47% of the genome in which, currently, ORFs have not been annotated. The confirmation of proteins corresponding to eleven novel ORFs and identification of ORF_59454 increases the number of experimentally-confirmed ASFV proteins to 154 across different isolates and genotypes. Functional characterizations of novel ORFs are essential to evaluate the biological role of the corresponding truncated proteins in the context of ASFV replication and virulence. Prime candidates for such experiments would include the nORFs detected with high consistency and shown in Table 1. The data concerning the comparison of expression kinetics on transcript and protein levels and the confirmation of new ORFs point out the importance of performing proteo-transcriptomic studies, to increase the understanding of gene expression and regulation in response to ASFV infection.

In general, the observed host responses after infection (with highly and moderately pathogenic ASFV-isolates, summarised in Table 2) were in line with described observations in vivo and in vitro regarding the expression and regulation of cytokines, antigen processing and presentation, and cell death. However, after infection with the moderately pathogenic Estonia 2014 isolate, we observed differential regulation of genes involved in cytokine responses and expression, MHC-mediated antigen processing, and presentation and cell death [15,27,28,29,31,89]. The expressions of some host genes were affected similarly after infection with either virus. The most prominent examples for this group were found among Cathepsins, which are known to be targeted during ASFV infection [3]. Other host genes, some of them involved in cell death and regulation of cytokine expression, were differentially impacted by the two isolates.

Even though it was postulated that caspase-mediated apoptosis played only a minor role during ASFV infection, ASFV encoded apoptosis antagonists such as pA179L and pA224L [89,90,91,92]. In the present study, we observed differential regulation of CASP8 between the highly and moderately pathogenic ASFV-isolates Armenia 2008 and Estonia 2014 at 6 hpi. While the expression of CASP8 was not significantly affected after infection with Estonia 2014 compared to mock-infected moMΦ at 6 hpi, it was no longer detected in moMΦ infected with Estonia 2014 at later time points, and was not detected after infection with Armenia 2008 at either time point. Additionally, the GO term “regulation of Caspases involved in apoptotic processes” (GO:0043281) was only enriched for proteins with significantly changed levels after infection with Estonia 2014 at 24 hpi.

CASP8 is involved not only in the regulation of programmed cell death mechanisms like apoptosis, necroptosis, and pyroptosis, but also in the regulation of the expression of inflammatory cytokines [93,94,95] such as pro-IL1ß [93]. Activation of CASP8 is tightly regulated by RIPK1 [94], which showed decreased expression levels in response to infection with either isolate. The unchanged expression of CASP8 in the moderately pathogenic isolate Estonia 2014 at early stages of infection could therefore provide a partial explanation for the less efficient suppression of cell death and cytokine expression, especially of IL1ß, described in the context of genotype I and II infections [29,89,96]. 

CASP8 is also involved in the regulation of pro-inflammatory gene expression mediated via stress-activated kinases JNK and p38 [93]. Currently, it remains unclear whether ASFV selectively targets one or several of the major MAPK signaling pathways. While effects on the expression of some kinases involved in MAPK signaling pathways have been noted after infection with highly pathogenic ASFV, the expression of others have remained unaffected [3,12]. In this study, our attention was drawn to several proteins involved in different MAPK signaling pathways. In line with previous observations [12], the expression of kinases belonging to the ERK signaling cascade (MAPK1, MAPK3, MAP2K1, and MAP2K2) remained unaltered after infection, while the levels of some proteins involved in MAPK14/p38 signaling changed significantly. These observations corresponded well with findings made for other DNA viruses such as Herpesvirus and polyomavirus, for which it has been described that, during infections, the MAPK/ERK function is performed via stress-activated MAPK/p38/JNK signaling pathways [97]. 

Both the phosphorylation at 6 hpi and the reduced expression levels of MAPK14/p38 at 24 hpi were induced by ASFV. The reduced expression levels appeared to be a result of ASFV-induced degradation processes rather than reduced synthesis, since transcription of p38 kinases appeared to be unaffected in response to ASFV infection and the protein half-lives, determined in different cell types including macrophage-progenitor cells monocytes, were longer than the experimental period analysed [12,98]. The observed modulation of the levels of MAP2K3/6 and MAPKAPK2, two genes that are highly specific for the MAPK14/p38 signaling pathway, confirmed that this pathway was affected during ASFV infection [99]. In contrast to MAPK14/p38, it seemed more likely that the synthesis rate of MAPKAPK2/3 was impaired during ASFV infection, based on decreased transcript levels reported in a transcriptome analysis of Georgia/07-infected macrophages [12].

Despite this fact, only minor effects of p38 activation or inhibition on viral replication and ASFV protein expression were observed, showing that ASFV was able to efficiently prevent detrimental effects of MAPK14/p38 activation during the early stages of infection. Furthermore, it remains possible that stress-activated kinase signaling and pro-inflammatory responses might also be mediated via JNK. This, unfortunately, was not detected in the current study. 

A transient upregulation of TAB1 and modulation of ZAP70 expression may have indicated the influence of ASFV infection on both the canonical and non-canonical MAPK14/p38 signaling pathways [99]. However, the ZAP70-path of activation is thought to be specific for T cells [99].

Within the canonical pathway, several receptors transmit their signals via MAPK14/p38. These include CD14, RIG-I-mediated signaling, GPCR-mediated signaling and TLR-mediated signaling [99]. Expression levels of all of these were affected during ASFV infection. RIG-I receptor signaling was enhanced in ASFV-Georgia-infected animals and identified as impaired during ASFV infection based on metabolite analysis in vitro [15,100]. Other in vitro analyses described the downregulation of CD14 and CXCR2, an IL8-receptor mediating its signal via G-proteins [12,37,101,102]. In addition to CD14, in the present study, the downregulation of TLR2 was observed. Interestingly, CD14 has been described to be critical for TLR2-mediated activation of inflammatory macrophages [103]. Furthermore, in the context of infections with other enveloped viruses, such as Herpes viruses and Vaccinia, the TLR2 heterodimer, using CD14 as ligand delivery in some cases, has been linked to T cell responses and increased production of TNFα and IL6 [104].

While it is known that ASFV inhibits TLR signaling events through reduced expression of multiple TLRs and antagonistic mechanisms mediated by pI329L and members of MGF 360 and MGF 505 [3,105,106,107], we described herein some possible indications for an additional mechanism of ASFV to inhibit TLR-mediated immune responses by inhibiting alternative signal transmission via MAPK14/p38.

Alternatively, MAPK14/p38 signaling can also be activated in response to ER stress, a known feature of ASFV infection [7,108,109]. In the present study, ER-stress response was identified by GO term enrichment analysis only in Armenia 2008-infected cells at 24 hpi. This could indicate differential interactions with the ER by highly and moderately pathogenic ASFV-isolates. Two members of MGF 110, which is absent from the Estonia 2014 genome, have been shown to locate in the ER [110,111]. Subsequent differences in the induction of ER stress and signaling events, also involving MAPK14/p38, may result in differential activation of cellular defence mechanisms in response to infection with viruses of different pathogenicity.

In addition to modulating the proinflammatory response, MAPK14/p38 is also involved in the control of the apoptotic machinery and macroautophagy via regulation of the phosphorylation of Bcl2-family members and Beclin-1 [99]. Interestingly, both members of the pro-apoptotic Bcl2 family and Beclin-1 interact with ASFV pA179L, a Bcl2-like protein expressed throughout the viral life cycle that has been described as a potent inhibitor of apoptosis and autophagy [2,5,92]. A179L, therefore, might also be involved in inhibiting effects induced in response to MAPK14/p38 activation during the early times of infection.

MAPK14/p38 activation has been described as inhibiting autophagy [112,113,114]. ASFV can inhibit the formation of autophagosomes in both Vero cells and primary macrophages at early times post-infection [5]. This observation could correspond to our observation of MAPK14/p38 phosphorylation and activation at 6 hpi induced by both highly and moderately pathogenic isolates. However, our data also indicated differences regarding the regulation of autophagy at later stages of infection. The term enrichment pointed at autophagy-related processes, like the development of Alzheimer’s disease, Huntington’s disease, and Parkinson’s disease [115], only in Armenia 2008-infected samples. The pathologies of all these neurodegenerative diseases have been associated with the activation of MAPK14/p38 and inhibition of the kinase had beneficial effects on the course of the disease [99].

## 5. Conclusions

Quantitative expression patterns of Armenia 2008 and Estonia 2014 ORFs, including some novel ORFs with alternative transcription start sites, were very similar after infection of primary monocyte-derived macrophages at early and late stages of infection. The analysis of existing structural models (p72) and modelling of pK78R and p150 suggested that the truncated ORFs might have biological functions divergent from those described for the full-length ORFs.

Analysis of the host proteome response to infection with one highly and one moderately pathogenic ASFV strain identified the MAPK14/p38 signaling pathway as a target of ASFV infection. Differential regulation of CASP8 and MAP2K3, two genes involved in the activation of MAPK14/p38, was observed, which could explain differences in the pro-inflammatory responses observed after infection with highly and less pathogenic ASFV isolates. Pathways that were differentially impacted by the two viruses related to immune response and regulation, antigen processing and presentation, and the response to and production of cytokines.

## Figures and Tables

**Table 1:** Identification of proteins corresponding to novel open reading frames (nORFs) [12].

nORF	Annotated ORF	# Unique Peptides	Detections (Replicates)				Kinetics
			Estonia 2014		Armenia 2008		
			6 hpi	24 hpi	6 hpi	24 hpi	
nORF_18417		1	3	-	2	-	early
nORF_176208	DP238L (e)	1	6	6	6	6	early
nORF_186513		2	3	3	1	1	early
nORF_125163	CP204L (e) *	2	3	4	2	1	early
nORF_180573		4	6	6	6	6	ambivalent
nORF_63288	K205R (e)	2	6	6	6	6	ambivalent
nORF_105178	B646L (l) *	2	-	5	-	6	late
nORF_143942		1	-	4	-	6	late
nORF_119520	CP2475L (l) *	1	-	5	-	5	late
nORF_63974	K78R (l) *	2	-	6	-	6	late
nORF_188532		1	-	-	-	2	late

(e): early; (l): late; * structural protein.

**Table 2:** Summary of the enrichment analysis of Kyoto Encyclopedia of Genes and Genomes (KEGG) pathways and biological processes in Gene Ontology (GO) terms (GO:BP) for up and downregulated genes compared to naïve monocyte-derived macrophages performed using the gProfiler multiquery option. Filled fields represent significant enrichments (black: *p*-value < 0.01; grey: *p*-value < 0.05; white: not significantly enriched, *p* ≥ 0.05).

Enriched GO and KEGG Terms and Descriptions	Armenia 2008		Estonia 2014	
	6 hpi	24 hpi	6 hpi	24 hpi
Phagosome KEGG:04145				
Lysosome KEGG:04142				
Citrate cycle (TCA cycle) KEGG:00020				
Autophagy related diseases KEGG:05010, KEGG:05014, KEGG:05016, KEGG:05012				
cell death GO:0008219, GO:0010941, GO:0012501, GO:0006915				
regulation of programmed cell death GO:0043067, GO:0042981				
intrinsic apoptotic signaling pathway GO:0097193				
regulation of intrinsic apoptotic signaling pathway GO:2001242				
positive regulation of apoptotic process GO:0043065, GO:2001235, GO:0010942, GO:2001238, GO:0043068, GO:2001236, GO:0043281				
Protein processing in endoplasmic reticulum KEGG:04141				
Spliceosome KEGG:03040, GO:0043484, GO:0048024				
endoplasmic reticulum to Golgi vesicle-mediated transport GO:0006888				
response to endoplasmic reticulum stress (ERAD pathway) GO:0036503, GO:0030433, GO:0034976				
Proteasome KEGG:03050				
immune response and regulation GO:0006955, GO:0002376, GO:0045087, GO:0050778, GO:0002684, GO:0050776, GO:0002682				
regulation of innate immune response GO:0045088				
Antigen processing and presentation KEGG:04612, GO:0019884, GO:0002478, GO:0002474, GO:0002504				
Response to and production of cytokines GO:0071345, GO:0001816, GO:0001817, GO:0034097, GO:0001819, GO:0023056				

## Data Availability

The mass spectrometry proteomics data have been deposited to the ProteomeXchange Consortium (http://proteomecentral.proteomexchange.org (accessed on 8 August 2022)) via the PRIDE partner repository (Perez-Riverol et al., 2019) with the dataset identifier PXD036402 and DOI 10.6019/PXD036402.

## Supplementary Materials

The following supporting information can be downloaded at: https://www.mdpi.com/article/10.3390/v14102140/s1, Figure S1: Workflow for data processing and statistical analysis performed in Perseus v1.6.15.0; Figure S2: Conformation of ASFV infection in cells prepared for MS analysis; Figure S3: Quantitative analysis of expression of viral proteins comparing 6 hpi and 24 hpi in Estonia or Armenia infected moMΦ; Figure S4: Detected peptides for nORFs that map to genome regions currently not annotated or represent novel N-termini in currently annotated ORFs; Figure S5: MS spectra of nORFs detected with only one unique peptide; Figure S6: Mapping of isoforms of nORF_59454, the corresponding aa sequences and the identified peptides to the ASFV-Georgia genome; Figure S7: Probability plot of structural prediction using Phobius of aa 1-842 of ASFV-p150 located N-terminal of the nORF_119520; Figure S8: Expression profiles of porcine genes during infection of moMΦ with Armenia 2008 or Estonia 2014 representing groups of host genes with specific expression patterns; Figure S9: Quantitative comparison of host protein expression levels of Armenia 2008 and Estonia 2014 infected moMΦ at 6 hpi and 24 hpi based on label-free quantification; Figure S10: Pathway enrichment and network analysis of selected host genes with outstanding host genes noted during the analysis of the host response to infection with Armenia 2008 and Estonia 2014; Figure S11: Impact of MAPK14/p38α on ASFV titers; Figure S12: Structure prediction of pK78R with AlphaFold; Table S1: Identifications and quantifications of ASFV and porcine proteins based on Maxquant analysis. (supplementary file); Table S2: Results of multiquery enrichment analysis of biological processes (GO:BP) and KEGG-pathways based on differentially expressed genes performed in gProfiler. (supplementary file); Table S3: List of selected porcine genes used for pathway enrichment and network analysis references to experimental observations in the present study that resulted in selection; Table S4: Results of enrichment, cluster and network analysis of genes listed in Table S3 using Cytoscape. (supplementary file).
