# Supplementary material for: Proteome Analysis of Swine Macrophages after Infection with Two Genotype II African Swine Fever Isolates of Different Pathogenicity
Source: Viruses. 2022 Sep 28;14(10):2140. doi: 10.3390/v14102140 (PMC9607119; doi:10.3390/v14102140)
Supplement: Supplementary file 1 [file viruses-14-02140-s001.zip › viruses-1932115-SI.pdf]

Supplementary Information

Title:

Proteome analysis of swine macrophages after infection with two genotype II African swine fever isolates of different pathogenicity

Authors:

Elisabeth Wöhnke, Gwenny Cackett, Finn Werner, Sandra Blome, Thomas C. Mettenleiter, Axel Karger

Supplementary Figures

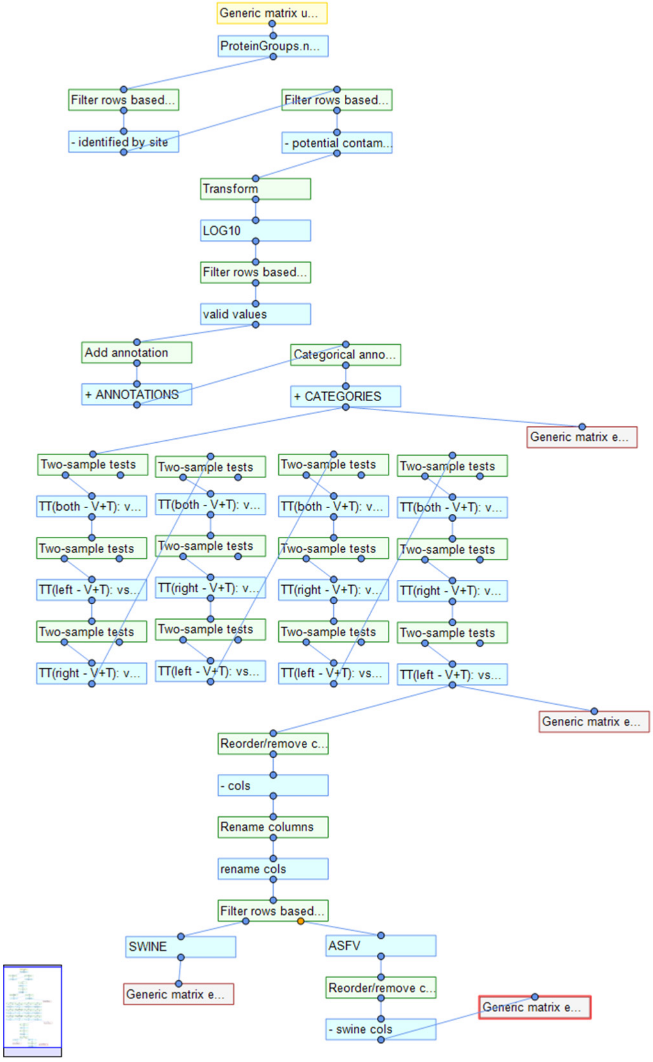

Figure S1: Workflow for data processing and statistical analysis performed in Perseus v1.6.15.0.

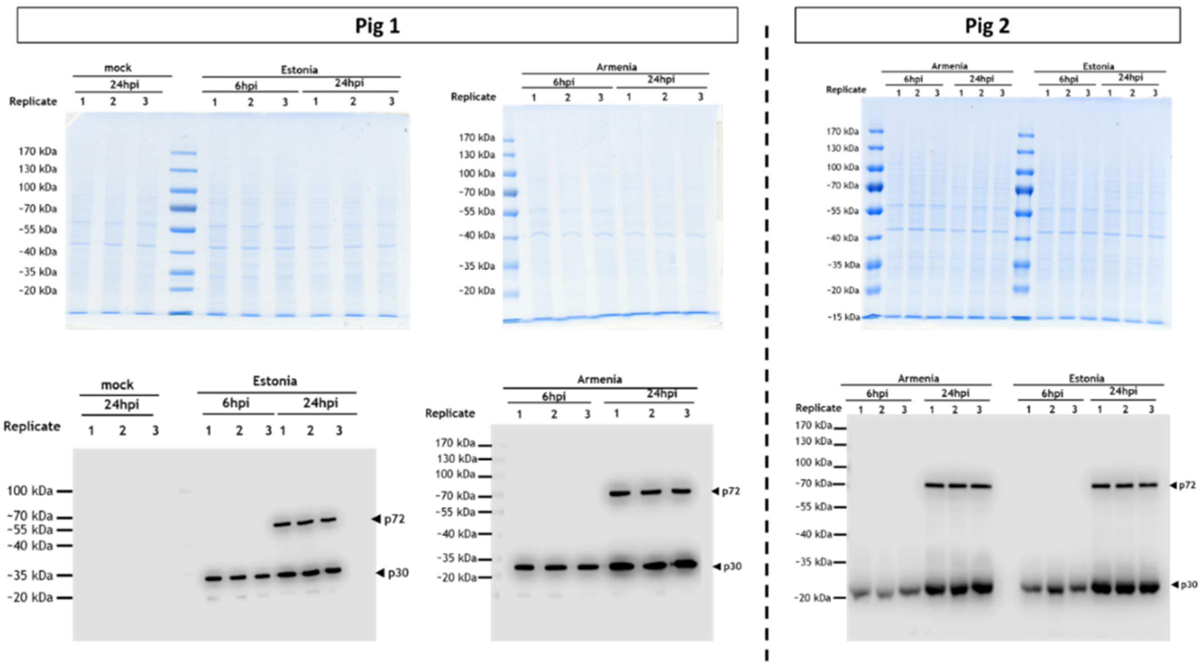

Figure S2: Confirmation of ASFV infection in cells prepared for MS analysis. Detection of p30 (early) and p72 (late) viral proteins in moMΦ in immunoblots (bottom) together with the corresponding Coomassie-stained SDS-PAGE gels (top).

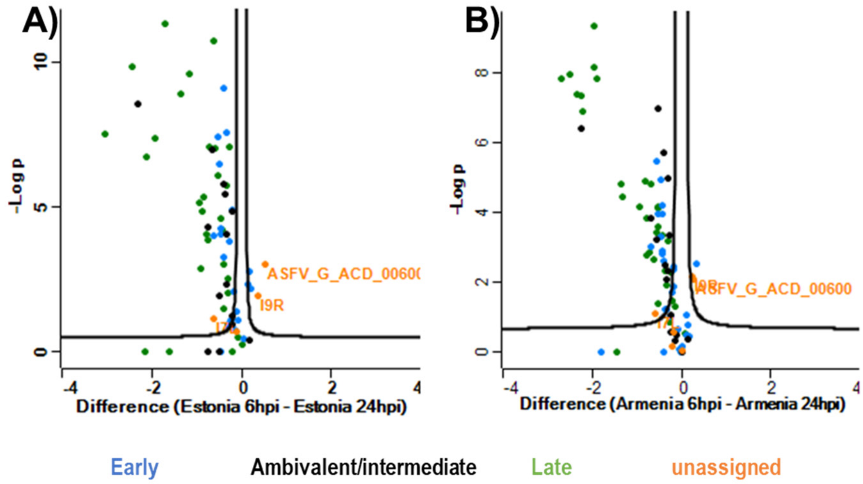

Figure S3: Quantitative analysis of expression of viral proteins comparing 6hpi and 24hpi in Estonia 2014 (A) or Armenia 2008 (B) infected moMΦ.

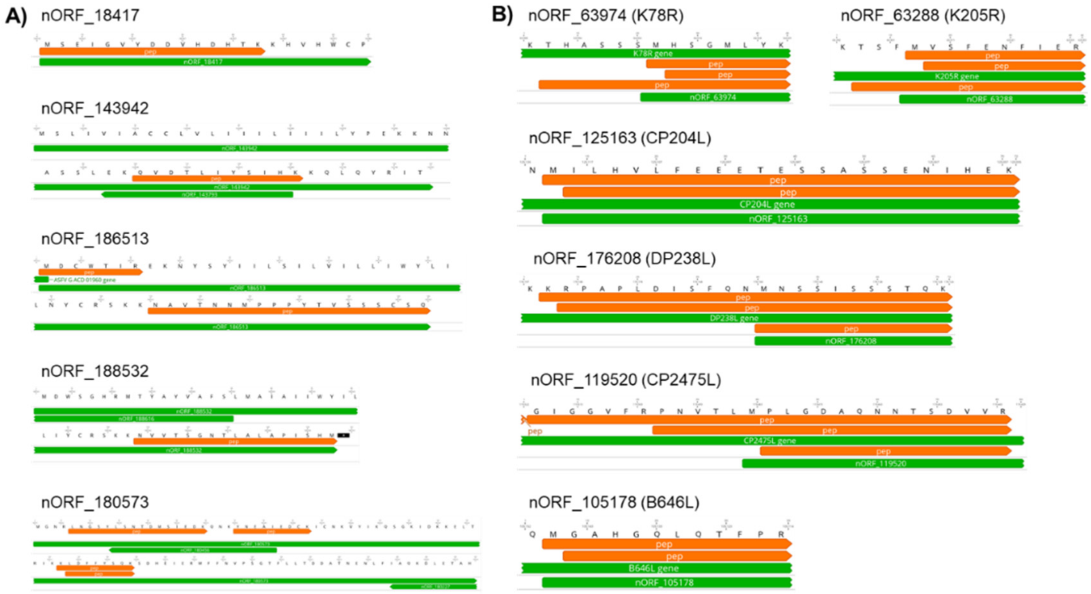

Figure S4: Detected peptides for nORFs that map to genome regions currently not annotated (**A**) or represent novel N-termini in currently annotated ORFs (**B**).

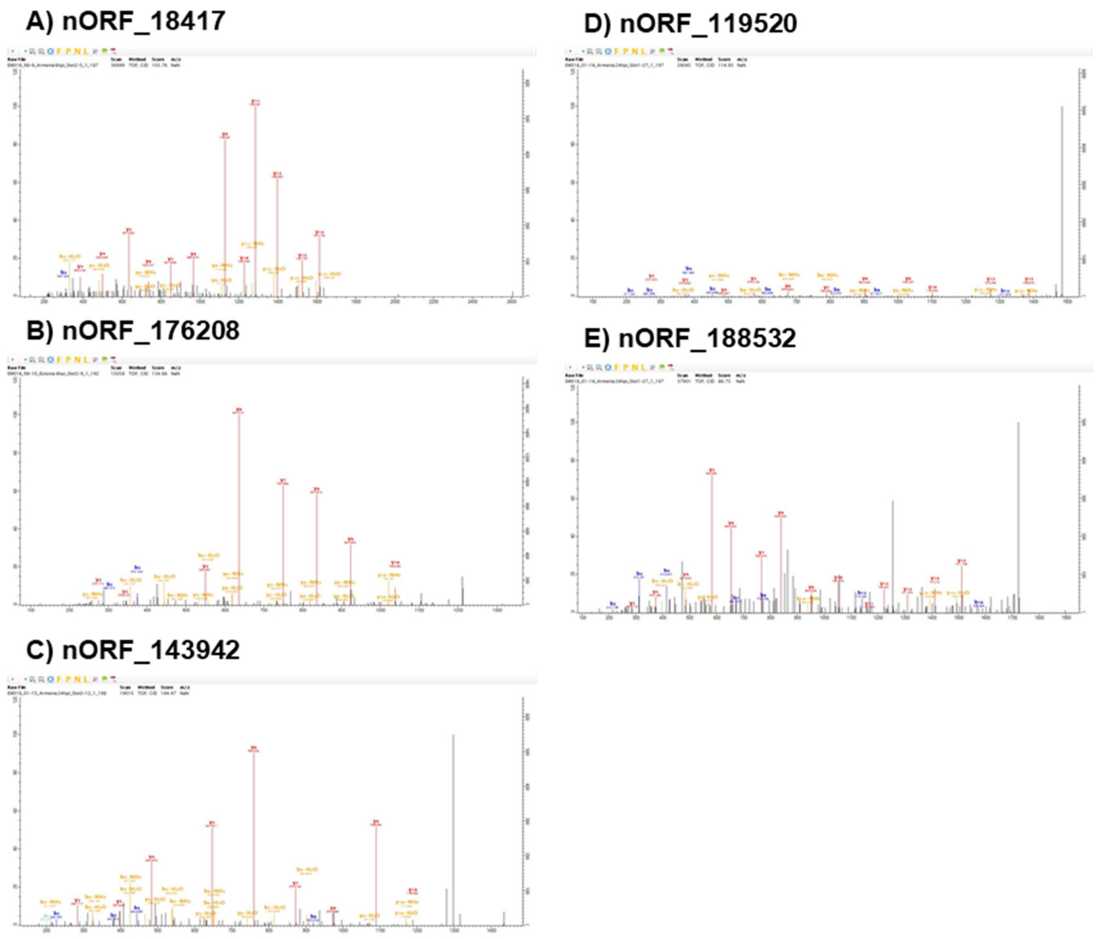

Figure S5: Fragment mass spectra of nORFs detected with only one unique peptide. Unique peptides were detected in at least two samples.

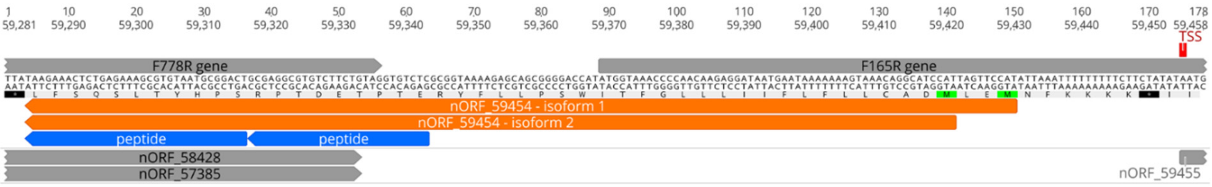

Figure S6: Mapping of isoforms of nORF_59454 (orange), the corresponding aa sequences and the identified peptides (blue) to the ASFV-Georgia genome. Translation start sites at positions 59 430 and 59421 are highlighted in green. Transcription start site (TSS) in red.

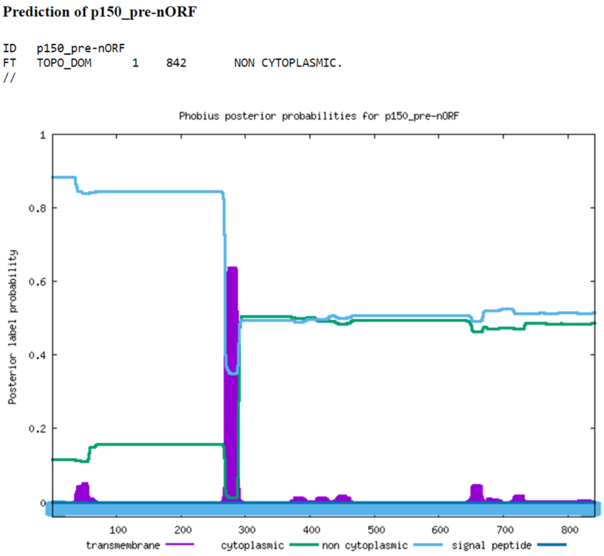

Figure S7: Probability plot of structural prediction using Phobius [1] of aa 1-842 of ASFV-p150 located N-terminal of the nORF_119520.

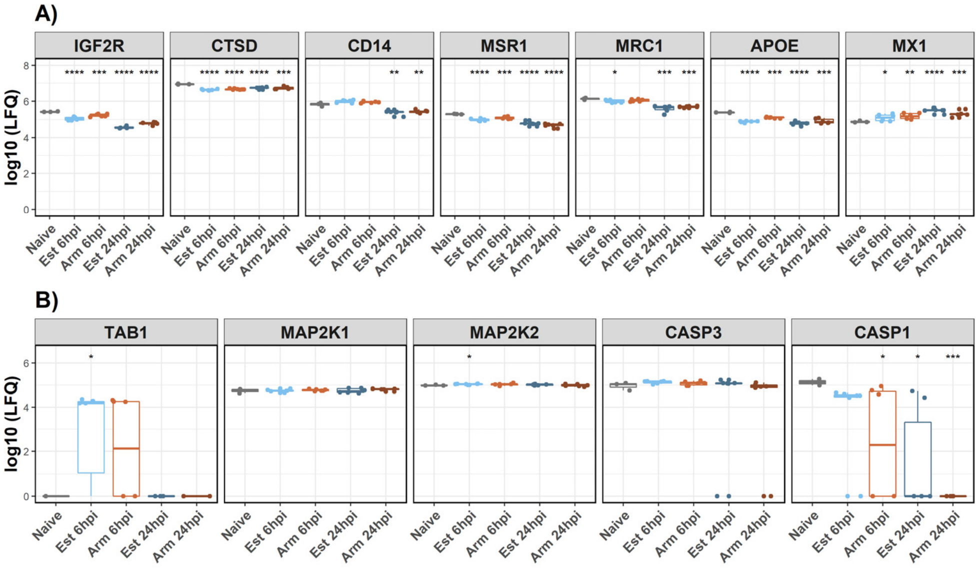

Figure S8: Expression profiles of porcine genes during infection of moMΦ with Armenia 2008 or Estonia 2014 representing groups of host genes with specific expression patterns.

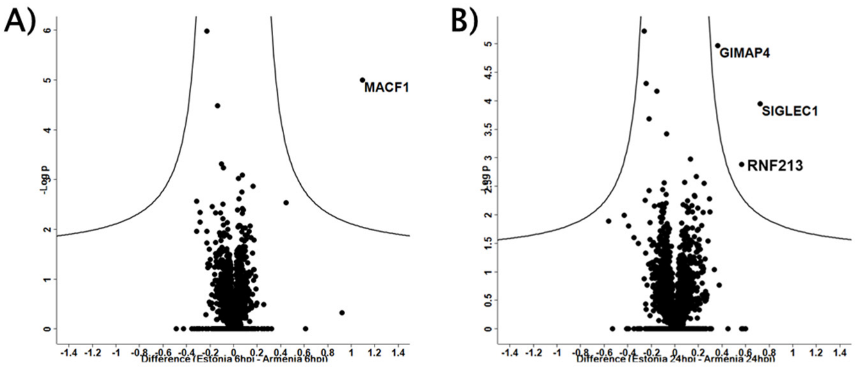

Figure S9: Quantitative comparison of host protein expression levels of Armenia 2008 and Estonia 2014 infected moMΦ at 6hpi (**A**) and 24hpi (**B**) based on label-free quantification. n = 6

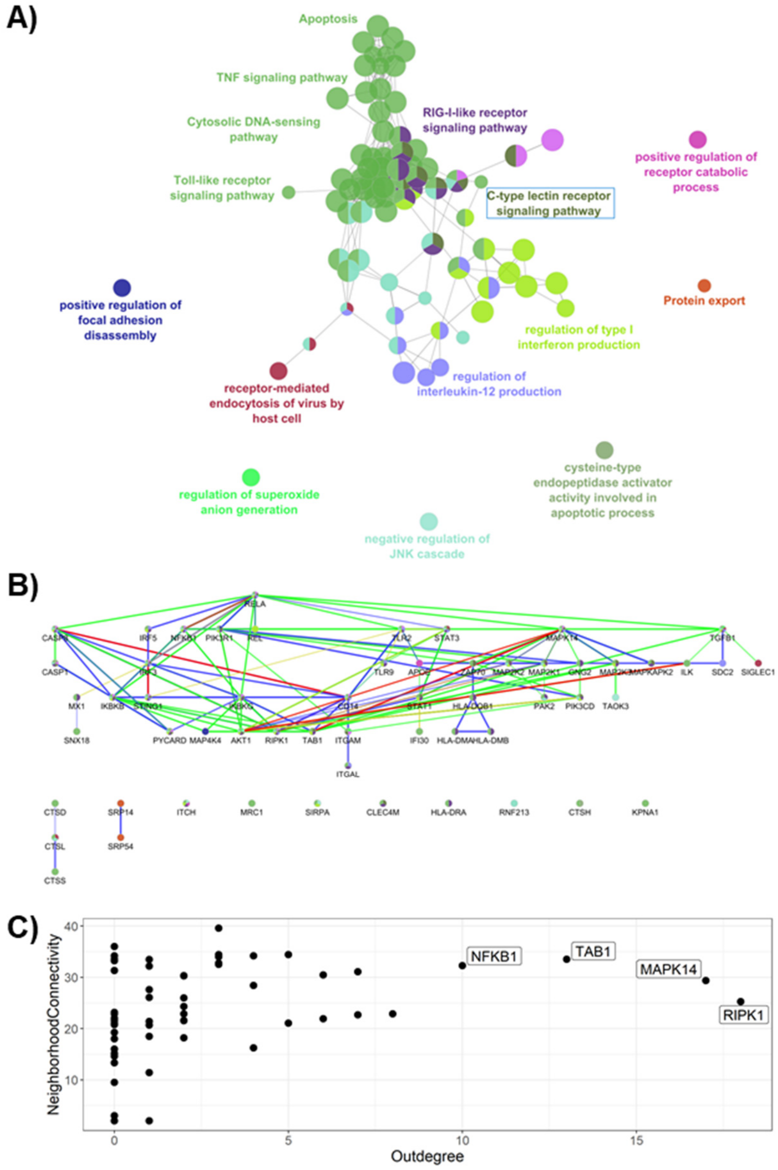

Figure S10: Pathway enrichment (**A**) and network analysis (**B**, **C**) of selected host genes with outstanding host genes noted during the analysis of host response to infection with Armenia 2008 and Estonia 2014 listed in Table S3 performed in Cytoscape using the plugins ClueGo [2] and CluePedia [3].

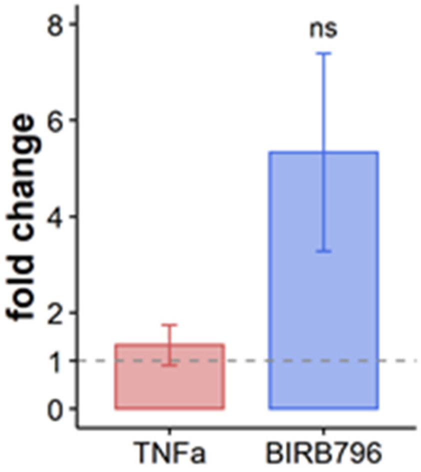

Figure S11: To assess the impact of p38 pre-stimulation or inhibition during infection with Armenia 2008, ASFV-titers of moMΦ treated with 20ng/mL TNFα for 4h or 25nM BIRB796 during the infection period were determined 24hpi and compared to untreated moMΦ. n = 3

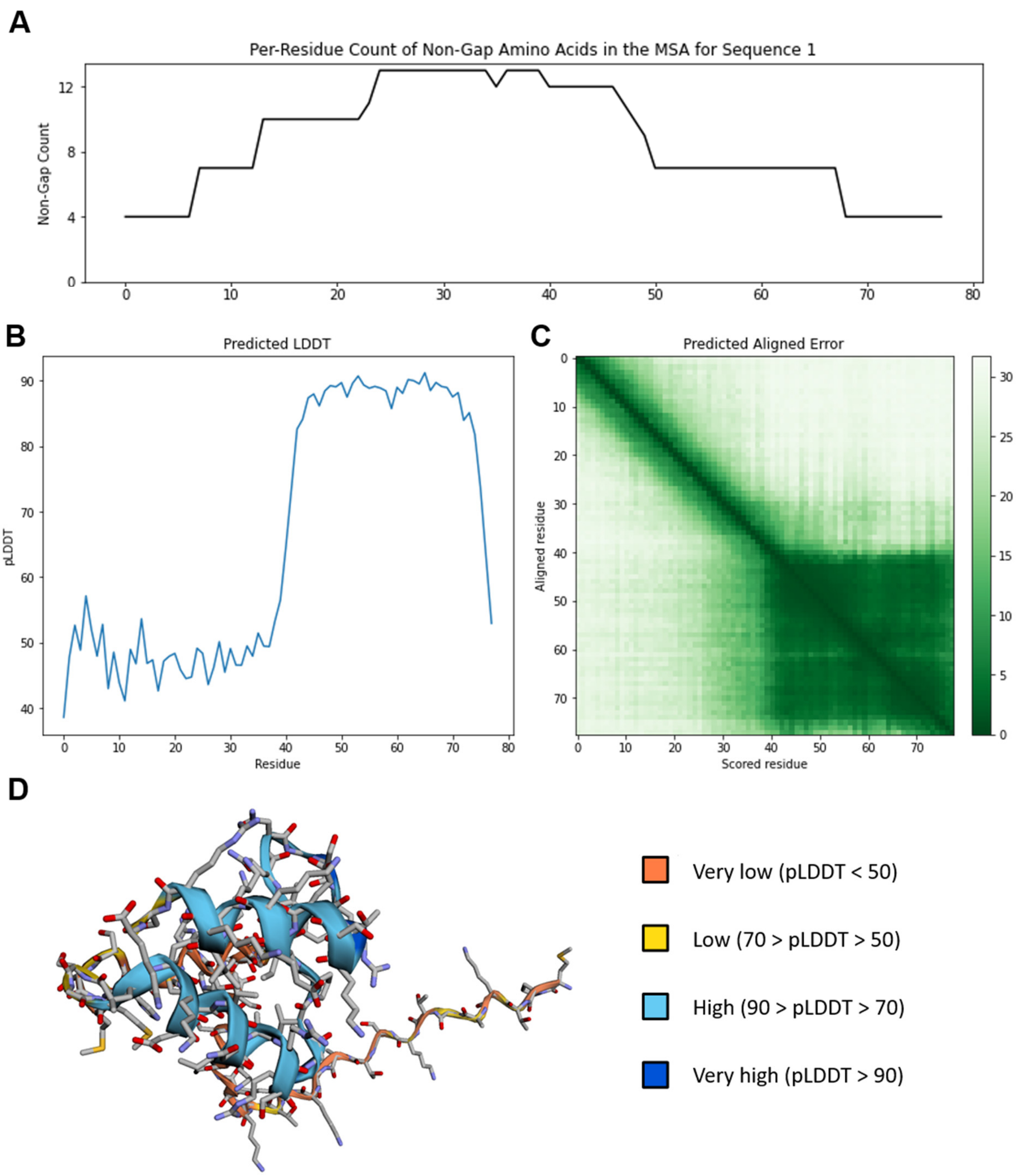

Figure S12 Structure prediction of pK78R with AlphaFold. (**A**) Multiple sequence alignment from running AlphaFold on pK78R, shown as sequences per position across the ORF sequence. (**B**) Predicted Local Distance Difference Test (pLDDT) across the sequence length of pK78R – lower pLDDT indicates lower confidence and likelihood of sequence being disordered. (**C**) Predicted Aligned Error across pK78R - for the relative position of predicted domains. (**D**) Per-residue pLDDT, as a colour scale on the model output of AlphaFold itself

Supplementary Tables

Supplementary File Table S1: Identifications and quantifications of ASFV and porcine proteins based on MQ analysis. A) Expression levels of ASFV proteins shown as log10 (LFQ) based on identifications of

annotated ORFs in Armenia 2008 (GenBank FR684268.2) and Estonia 2014 genomes (GenBank LS478113). Results of statistical testing and detected acetylated protein N-termini based on MQ results. B) Identified peptides annotating within nORFs predicted by Cackett et al., 2022. Proteins, Leading razor protein, peptide sequence, score, intensity and detection of acetylated protein N-termini based on MQ analysis. C) Expression levels of porcine proteins are shown as log10 (LFQ). Porcine protein identifiers (majority protein IDs) were converted into HGNC nomenclature. Results of statistical testing and detected acetylated protein N-termini based on MQ results.

Supplementary File Table S2: Results of multiquery enrichment analysis of Gene Ontology biological processes (GO:BP) and KEGG-pathways based on differentially expressed genes performed in gProfiler. Significantly enriched terms are highlighted in bold-black. Term and group-specific p-values are shown rounded to 4 digits.

Table S3: List of selected porcine genes used for pathway enrichment and network analysis references to experimental observations in the present study that resulted in selection.

Gene	Reference	Gene	Reference	Gene	Reference
AKT1	Figure 4	ITGAM	Table S1C	SRP14	Figure 4
APOE	Table S1C	KPNA1	Table S1C	SRP54	Figure 4
CASP1	Table S1C	LAMTOR4	Figure 4	STAT1	Table S1C
CASP8	Figure 4	MACF1	Figure S9	STAT3	Table S1C
CD14	Table S1C	MAP2K2	Table S1C	STING1	Figure 4
CLEC4M	Table S1C	MAP2K3	Figure 4	STK10	Figure 4
CLINT1	Figure 4	MAP4K4	Figure 4	STK26	Figure 4
CLIP1	Table S1C	MAPK14	Table S1C	STK38L	Figure 4
CTSA	Table S1C	MAPK8IP3	Figure 4	STX6	Figure 4
CTSD	Table S1C	MAPKAPK2	Figure 4	TAB1	Figure 4
CTSH	Table S1C	MRC1	Table S1C	TAOK3	Figure 4
CTSL	Table S1C	MSR1	Table S1C	TGFB1	Figure 4
CTSS	Table S1C	MX1	Table S1C	TGFBI	Figure 4
CTZ	Table S1C	PAK2	Table S1C	TLR2	Table S1C
GIMAP4	Figure S9	PIK3CD	Figure 4	TLR9	Table S1C
GIMAP8	Figure 4	PIK3R1	Figure 4	TNFAIP2	Figure 4
GNG2	Figure 4	PLXNC1	Table S1C	UBA3	Table S1C
HLA-DMA	Table S1C	POLDIP3	Table S1C	UBA5	Table S1C
HLA-DMB	Table S1C	PPP1CC	Figure 4	UBE3A	Table S1C
HLA-DQB1	Table S1C	PSMB7	Figure 4	UBE3C	Table S1C
IGF2R	Figure S9	PTK6	Table S1C	UBL5	Figure 4
IKBKB	Figure 4	PYCARD	Figure 4	UBR4	Figure 4
IKBKG	Figure 4	RIPK1	Table S1C	USP24	Figure 4
ILK	Table S1C	RNF213	Figure S9	VPS35	Table S1C
IRF3	Table S1C	SDC2	Figure 4	VPS37A	Figure 4
IRF5	Table S1C	SIGLEC1	Figure S9	VPS51	Figure 4
ITBG1	Table S1C	SIRPA	Table S1C	VPS52	Figure 4
ITCH	Figure 4	SNX17	Table S1C	ZAP70	Figure 4
ITGAL	Table S1C	SNX18	Figure 4		

Supplementary File Table S4: Results of enrichment, cluster and network analysis of genes listed in Table S3 using Cytoscape.

1. Kall, L.; Krogh, A.; Sonnhammer, E. L., A combined transmembrane topology and signal peptide prediction method. *Journal of molecular biology* **2004,** 338, (5), 1027-36.
2. Bindea, G.; Mlecnik, B.; Hackl, H.; Charoentong, P.; Tosolini, M.; Kirilovsky, A.; Fridman, W.-H.; Pagès, F.; Trajanoski, Z.; Galon, J., ClueGO: a Cytoscape plug-in to decipher functionally grouped gene ontology and pathway annotation networks. *Bioinformatics (Oxford, England)* **2009,** 25, (8), 1091-1093.
3. Bindea, G.; Galon, J.; Mlecnik, B., CluePedia Cytoscape plugin: pathway insights using integrated experimental and in silico data. *Bioinformatics (Oxford, England)* **2013,** 29, (5), 661-663.
